# The SeqWord Genome Browser: an online tool for the identification and visualization of atypical regions of bacterial genomes through oligonucleotide usage

**DOI:** 10.1186/1471-2105-9-333

**Published:** 2008-08-07

**Authors:** Hamilton Ganesan, Anna S Rakitianskaia, Colin F Davenport, Burkhard Tümmler, Oleg N Reva

**Affiliations:** 1 Dep. of Biochemistry, Bioinformatics and Computational Biology Unit, University of Pretoria, Lynnwood road, Hillcrest, Pretoria, 0002, South Africa; 2 Klinische Forschergruppe, OE 6710, Medizinische Hochschule Hannover, D-30625 Hanover, Germany

## Abstract

**Background:**

Data mining in large DNA sequences is a major challenge in microbial genomics and bioinformatics. Oligonucleotide usage (OU) patterns provide a wealth of information for large scale sequence analysis and visualization. The purpose of this research was to make OU statistical analysis available as a novel web-based tool for functional genomics and annotation. The tool is also available as a downloadable package.

**Results:**

The SeqWord Genome Browser (SWGB) was developed to visualize the natural compositional variation of DNA sequences. The applet is also used for identification of divergent genomic regions both in annotated sequences of bacterial chromosomes, plasmids, phages and viruses, and in raw DNA sequences prior to annotation by comparing local and global OU patterns. The applet allows fast and reliable identification of clusters of horizontally transferred genomic islands, large multi-domain genes and genes for ribosomal RNA. Within the majority of genomic fragments (also termed genomic core sequence), regions enriched with housekeeping genes, ribosomal proteins and the regions rich in pseudogenes or genetic vestiges may be contrasted.

**Conclusion:**

The SWGB applet presents a range of comprehensive OU statistical parameters calculated for a range of bacterial species, plasmids and phages. It is available on the Internet at .

## Background

The study of genome OU signatures has a long history dating back to early publications by Karlin *et al*. who focused mainly on dinucleotide compositional biases and their evolutionary implications [1-3]. Statistical approaches of OU comparison were further advanced by Deschavanne *et al*., who applied chaos game algorithms [4]; and by Pride *et al*., who extended the analysis to tetranucleotides using Markov Chain Model simulations [5]. Later, a number of practical tools for phylogenetic comparison of bacterial genomes [4,6,7], identification of horizontally transferred genomic islands [8-13] and assignment of unknown genomic sequences [14,15] based on OU statistics became publicly available. These approaches exploited the notion that genomic OU composition was less variable within genomes rather than between them, regardless of which genomic regions had been taken into consideration [16]. A general belief was that if a significant compositional difference was discovered in genomic fragments relative to the core genome, these loci most likely can be assigned to horizontally transferred genetic elements (transposons, prophages or integrated plasmids). This approach was criticized by several researchers [17,18] who pointed out that codon bias and base composition are poor indicators of horizontal gene transfer. Therefore, there is a need for more informative parameters which also take into account higher order DNA variation. An overview of the current OU statistical methods based on di-, tetra- and hexanucleotides has been published recently. The conclusion of the review was that all methods were context dependent and, though being efficient and powerful, none of them were superior in all applications [19]. Thus, the major motivation of our work was to develop more flexible and informative algorithms seamlessly integrating di- to heptanucleotides OU analysis for reliable identification of divergent genomic regions.

Recently we have introduced the concept of OU patterns into the literature [20]. Each OU pattern is characterized by a number of OU statistical parameters namely, local pattern deviation (D), pattern skew (PS), relative variance (RV) and others (see Methods section). Novelties of the developed algorithms relative to other existing methods include the following: i) distances between patterns of different word length (from di- through to heptanucleotides) calculated for the same sequences are comparable; i.e. one may use longer word patterns to perform a large scale analysis and then switch to shorter word patterns for a more detailed view; ii) OU patterns calculated for sequences of different lengths are comparable provided that the length of the sequence is longer than the corresponding thresholds (specified in the Methods section); iii) alterations of OU patterns may be analyzed by different non-redundant parameters (D, PS and RV with different schemes of normalization by frequencies of shorter constituent words). Superimposition of these OU characteristics allows better discrimination of divergent genomic regions relative to other contemporary approaches [21].

## Implementation

Calculation of OU statistical parameters has been described previously [20,21]. OU pattern was denoted as a matrix of deviations Δ_[*ξ*1...*ξN*] _of observed from expected counts for all possible words of length *N*:

(1) Δ_[*ξ*1...*ξN*] _= (*C*_ [*ξ*1...*ξN*]|*obs *_- *C*_ [*ξ*1...*ξN*]|*e*_)/*C*_ [*ξ*1...*ξN*]|0_

where *ξ*_n _is any nucleotide A, T, G or C in the *N*-long word; *C*_ [*ξ*1...*ξN*]|*obs *_is the observed count of the word [*ξ*_1_...*ξ*_N_]; *C*_ [*ξ*1...*ξN*]|*e *_is the expected count and *C*_ [*ξ*1...*ξN*]|0 _is a standard count estimated from the assumption of an equal distribution of words in the sequence: (*C*_ [*ξ*1...*ξN*]|0 _= *L*_*seq *_× 4^-*N*^).

Expected counts of words *C*_ [*ξ*1...*ξN*]|*e *_were calculated in accordance with the applied normalization scheme. Thus, *C*_ [*ξ*1...*ξN*]|*e *_= *C*_ [*ξ*1...*ξN*]|0 _if OU is not normalized, or *C*_ [*ξ*1...*ξN*]|*e *_= *C*_ [*ξ*1...*ξN*]|*n *_if OU is normalized by empirical frequencies of all shorter words of the length *n*. The expected count of a word *C*_ [*ξ*1...*ξN*]|*e *_of length *N *in a *L*_*seq *_long sequence normalized by frequencies of *n*-mers (*n *<*N*) was calculated as follows:

(2) $$C_{[\xi_{1}...\xi_{l_{w}}]|n}=L_{seq}\times F_{\left[\xi_{1}...\xi_{n}\right]}\times \prod_{i=2}^{N-n+1}\left(\frac{F_{[\xi_{i}...\xi_{i+n-1}]\xi_{i+n}}}{\sum_{\xi }^{A,T,G,C}F_{[\xi_{i}...\xi_{i+n}]\xi }}\right)$$

where the *F*_ [*ξ*1...*ξn*] _values are the observed frequencies of the particular word of length *n *in the sequence and *ξ *is any nucleotide A, T, G or C. For example, expected count of a word ATGC in a sequence of *L*_*seq *_nucleotides normalized by frequencies of trinucleotides is:

(3) $$C_{ATGC}=L_{seq}\times F_{ATG}\times \frac{F_{TGC}}{F_{TGA}+F_{TGT}+F_{TGG}+F_{TGC}}$$

Two approaches of normalization have been exploited where the *F *values were calculated for the complete sequence of a chromosome, plasmid, etc (generalized normalization) or for a given sliding window (local normalization). The normalization by equation 2 allows identification of words, frequencies of which cannot be predicted exactly by frequencies of shorter constituent words.

The distance *D *between two patterns was calculated as the sum of absolute distances between ranks of identical words (*w*, in a total 4^*N *^different words) after ordering of words by Δ_[*ξ*1...*ξN*] _values (see equation 1) in patterns *i *and *j *as follows:

(4) $$D(\%)=100\times \frac{\sum_{w}^{4^{N}}\left|rank_{w,i}-rank_{w,j}\right|-D_{\min}}{D_{\max}-D_{\min}}$$

Application of ranks instead of relative oligonucleotide frequency statistics made the comparison of OU patterns less biased to the sequence length provided that the sequences are longer than the limits of 0.3, 1.2, 5, 18.5, 74 and 295 kbp for di-, tri-, tetra-, penta-, hexa- and heptanucleotides, respectively [20].

PS is a particular case of D where patterns *i *and *j *were calculated for the same DNA but for direct and reversed strands, respectively. D_max _= 4^*N *^× (4^*N *^- 1)/2 and D_min _= 0 when calculating a D or, in a case of PS calculation, D_min _= 4^*N *^if *N *is an odd number or D_min _= 4^*N *^- 2^*N *^if *N *is an even number due to presence of palindromic words [20]. Normalization of D-values by D_max _ensures that the distances between two sequences are comparable regardless of the word length of OU patterns.

Relative variance of an OU pattern was calculated by the following equation:

(5) $$RV=\frac{\sum_{w}^{4^{N}}\Delta_{w}^{2}}{\left(4^{N}-1\right)\sigma_{0}^{2}}$$

where N is word length; Δ^2^_*w *_is the square of a word *w *count deviation (see equation 1); and *σ*^2^_0 _is the expected variance of the word distribution in a randomly generated sequence that depends on the sequence length and the word length:

(6) $$\sigma_{0}^{2}=0.14+\frac{4^{N}}{L_{seq}}$$

where *L*_*seq *_is sequence length, and N is word length. Normalization of OU pattern variance by *σ*_0 _makes the variances comparable regardless of the word length of OU patterns and the sequence length. The regression equation was tested on 300 randomly generated sequences with an equiprobable occurrence of all 4 nucleotides by the DataFit 7.1.44 software.

The SWGB is coded in Java to be used as an applet in a Web-browser either on the Internet or locally (the programs OligoWords in Python and SeqWord_Viewer, which respectively calculate and visualize the OU patterns for DNA sequences, are available for download from the SWGB website). SWGB should run on any platform with a Java 1.5.x runtime environment or newer.

The pre-calculated data-sets are saved in a MySQL Server 5.0 database. The size of the sliding window and the OU pattern type were applied according to the sequence length (Table 1). At the time of writing, the SeqWord database contained OU patterns pre-calculated for the sequences of 682 bacterial chromosomes belonging to 637 different organisms (strains and species), 412 plasmids, 100 bacteriophages and 39 other viruses, which were downloaded from the NCBI [44].

**Table 1:** Sliding window size and OU pattern types (oligomer lengths) selected for sequences of different length present in the SeqWord database.

**Sequence length**	**Sliding window**	**Step**	**OU pattern type**
> 2 Mbp	8 kbp	2 kbp	4 mer
from 1 mbp to 2 Mbp	5 kbp	0.5 kbp	4 mer
from 0.5 mbp to 1 Mbp	3 kbp	0.3 kbp	3 mer
< 0.5 Mbp	1.5 kbp	0.15 kbp	3 mer

## Results

User familiarity with the abbreviations of the various OU statistical parameters is important. Different types of OU patterns were abbreviated as type_*N*mer. Types might be "n0" for non-normalized, or "n1" for normalized by mononucleotide frequencies. For example, the non-normalized tetranucleotide usage pattern is denoted as n0_4mer; tetranucleotide usage pattern normalized by mononucleotide content is n1_4mer etc. The genomes in the SWGB database were analyzed by the following statistical parameters: D – distance between two patterns of the same type (in this work we used distances D between local patterns calculated for overlapping genome fragments and the global genome patterns calculated for the complete sequence – the local pattern deviation); PS – pattern skew, distance between the two patterns of the direct and reverse strands of the same DNA sequence; RV and GRV – oligonucleotide usage variances normalized locally and globally, respectively, and reduced to the OU variance expected for a randomly generated sequence (see Materials section); GC-content (GC) and GC-skew (GCS) in DNA fragments.

### The applet GUI and database of pre-calculated OU patterns

The SeqWord Genome Browser (SWGB) applet is available via the Internet [22-24] and is mouse and menu driven. The Web-based applet is used to visualize DNA compositional variations in bacterial and viral genomes stored in the SeqWord database. Every genome in the database is represented by a set of statistical OU parameters (D, PS, GV, GRV, GC and GCS) calculated for genomic fragments, which were selected by a sliding window (sliding window length and step were set according to the total length of the sequence as demonstrated in Table 1). While in 70 to 99% of genomic fragments the OU compositional bias is similar to the complete genome OU pattern, some regions with atypical OU composition, however, are always present. Superimposition of different OU parameters allows discrimination of divergent genomic regions, as was published previously [21]. Briefly: rRNA operons are characterized by extremely high PS and low RV; giant genes with multiple repeated elements have high or moderate PS and high RV; horizontally transferred genetic elements are characterized by increased divergence between RV and GRV accompanied by high D; and genes for ribosomal proteins show a moderate increase of D, PS and RV above genomic averages. Having analyzed 1243 sequences of different microorganisms including viruses and plasmids in the SeqWord database, we confirmed that the approaches we have developed and tested previously [25] (mainly on *Pseudomonas putida *KT2440 chromosomal DNA) are appropriate and useful for analysis of genomic sequences of other microorganisms and viruses.

**Figure 1:**
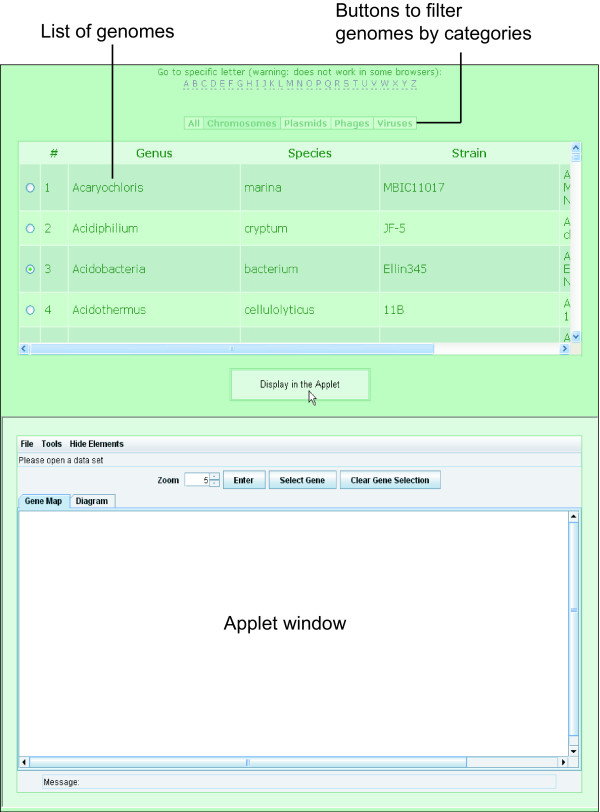
**General view of the web-based SWGB with a list of genomes present in the database and an enclosed Java applet for data visualization**. To show OU statistical parameters for a selected genome, click the 'Display in the Applet' button. Click a filter button to order genomes by the corresponding category and use the interactive letters at the top to scroll the list to a sequence of interest.

In an open applet window, the user has the ability to choose from an ever growing list of available sequences (Fig. 1). The user also has the option of restricting the list to display only bacterial chromosomes, plasmids, phages, viruses or all sequences by selecting the corresponding filter button. Users have to select a genome in the list and click the 'Display in the Applet' button to retrieve the pre-calculated data. All OU parameters calculated for a given genome may be exported to a local text file by using the 'Export' function from the applet's 'File' menu. Later, instead of again having to connect to the database, users may open and view their local files (previously exported from the applet or calculated by the OligoWords program, see below) via the 'Open' function in the 'File' menu.

The SWGB is basically comprised of two views, denoted by the 'Gene Map' and 'Diagram' tabs. The applet is instrumental for visualization of natural variation in DNA sequences by the interactive diagrams on the 'Gene Map' and 'Diagram' tabs. Users may save the current diagram in JPG format by using the 'Save picture' function in the 'File' menu.

**Figure 2:**
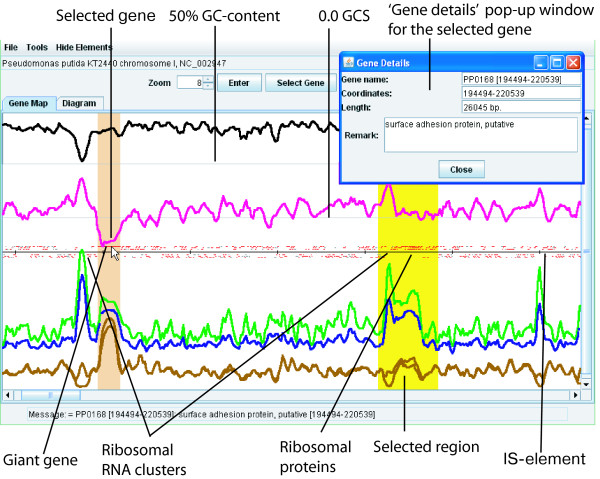
**Identification of divergent genomic regions on the 'Gene Map' view**. Superimposition of different OU parameters such as GC (black line), GCS (pink), PS (green), D (blue), GRV (upper brown line) and RV (lower brown line) allows discrimination of divergent genomic regions. In this example a part of the chromosome of *Pseudomonas putida *KT2440 (127–774 kbp) is displayed in the applet window. A genomic fragment was highlighted using the function 'Select region' and a giant gene, PP0168, was selected by 'Select gene'. A pop-up window 'Gene Details' was opened by double-clicking the gene on the map. Genes are indicated by red and grey (for hypotheticals) bars. The black horizontal line separates genes by their direction of translation.

The 'Gene Map' tab offers a simple view of an entire genome at a glance and gives users access to a number of important pre-calculated OU statistics superimposed on the gene map (Fig. 2). Displays for each of the statistical parameters can be toggled on/off by checking items in the 'Hide Elements' menu. By merely mousing over any region on the plot, a message displaying detailed information for the pointed curve will be shown in the 'Message' bar. Clicking a gene on the map displays a dialog with the annotation details (Fig. 2).

The 'Zoom' function is straight-forward and allows users to control the amount of data viewed in the plot area. Clicking the 'Enter' button after setting the desired zoom value will then redraw the map. A 'Zoom into region' function under the 'Tools' drop-down menu allows users to zoom into exact genomic regions by merely entering their desired co-ordinates into the pop-up dialog box. The 'Tools' → 'Select region' menu item allows highlighting of selected regions without zooming. Use the option 'Clear ...' in the 'Tools' menu to undo zooming or highlighting. To locate a genomic region by gene, click the button 'Select Gene'. In the pop-up dialog box one may order the gene list by gene names, functionality or coordinates, then select a gene in the list and click 'OK'. When a gene annotation is not available, the values of the locus coordinates are used as a gene name. The applet window will be scrolled to the selected gene highlighted on the map (see Fig. 2).

The 'Diagram' tab allows flexible filtering of the underlying data based on the criteria chosen by users. Although the underlying data is pre-calculated, the user may, by simply changing selected parameters, generate very different images which give different insights into the natural genomic variation. To start with, the 'Diagram' view offers a bar chart or a dot-plot presentation of the pre-calculated data. To view a bar chart of the distribution statistics for a given OU parameter, select the desired parameters from the X or Y-axis drop-downs and click 'Enter'. The number of bars displayed can be adjusted using the '# Bars' selector.

**Figure 3:**
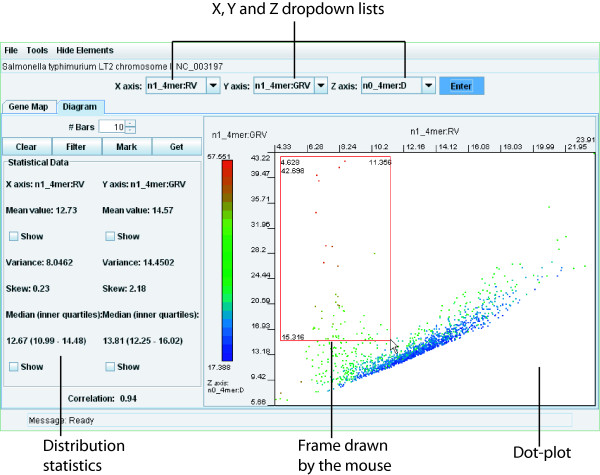
**The 'Diagram' view**. To draw a diagram, first select corresponding OU parameters using the dropdown lists and click the 'Enter' button. In this example n1_4mer:RV, n1_4mer:GRV and n0_4mer:D were selected for the X, Y and Z axes, respectively. Every dot on the dot-plot corresponds to a genomic fragment selected by the sliding window. Dots are spread and coloured in accordance with their values of the selected statistical OU parameters. Information for each dot may be found by one of the following methods: i) information for a dot under the mouse pointed by the mouse is shown in the 'Message' bar; ii) double clicking a dot returns us to the 'Gene map' tab with the corresponding genomic fragment highlighted; iii) framing the dots and clicking the 'Get' button opens a new applet window with the information about all selected regions. In this example the genomic regions of *Salmonella typhimurium *LT2 (NC_003197) that correspond to horizontally transferred genetic elements were selected (see discussion in the text).

On the dot-plot diagram, each genomic fragment (selected by the sliding window) is represented by a dot with X and Y coordinates that correspond to values of OU parameters chosen from X and Y drop-down lists, respectively. The Z axis parameter may be set as well. In this case, the dots are coloured by values of OU parameters selected for the Z axis, and the colour range is displayed on the vertical colour bar on the left of the plot area (Fig. 3).

Having set up the dot-plot, users will be able to identify divergent genomic regions (see next section). To retrieve annotations of genomic fragments corresponding to a group of dots, frame the dots of interest by clicking and dragging over the desired area. A selector frame then appears around the dots (Fig. 3). Clicking the 'Get' button displays the selected genomic fragments with their coordinates and gene annotations. Furthermore, identification and isolation of specific genomic regions may be improved significantly by filtering dots by OU parameters. The simplest way of filtering is by the third (Z axis) parameter. One may select an area on the colour bar to exclude all dots from the plot lying outside of the selected colour range (see an example in help files on-line). The hidden dots will not be selected by the 'Get' button. A more sophisticated way to filter genomic regions is provided by the 'Filter' button. An example will be discussed below.

**Figure 4:**
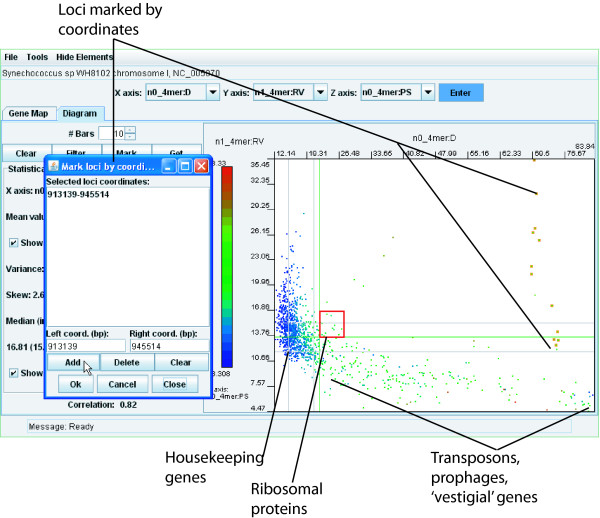
**Identification of divergent genomic regions by plotting and highlighting**. In this example the genome of *Synechococcus *sp. WH8102 was analysed. The parameters n0_4mer:D, n1_4mer:RV and n0_4mer:PS were selected for the X, Y and Z axes, respectively. The genomic regions covering the giant gene for the surface protein SwmB [29] were highlighted by entering the coordinates of this gene into the 'Mark loci by coordinates' dialog. The genomic regions enriched with i) housekeeping genes; ii) genes for ribosomal proteins; iii) vestigial genetic elements (comprising pseudogenes, transposons, prophages and IS-elements) are indicated.

The 'Mark' button enables genomic fragments to be selected by their coordinates and highlighted on the dot-plot. Click the 'Mark' button to open a dialog and enter coordinates of one or multiple fragments (Fig. 4). Co-ordinates of each fragment must be added to the list by clicking the 'Add' button. Close the dialog by clicking 'OK'. The corresponding dots on the dot-plot will be highlighted as shown in Fig. 4.

### Identification of divergent genomic islands

Several routines have been developed to identify the horizontally transferred genomic islands, genes for ribosomal RNA and proteins, non-functional pseudogenes and genes of other functional categories. All these routines are described in detail with illustrations in supplementary web-pages (use the 'Help' link in the applet window).

**Figure 5:**
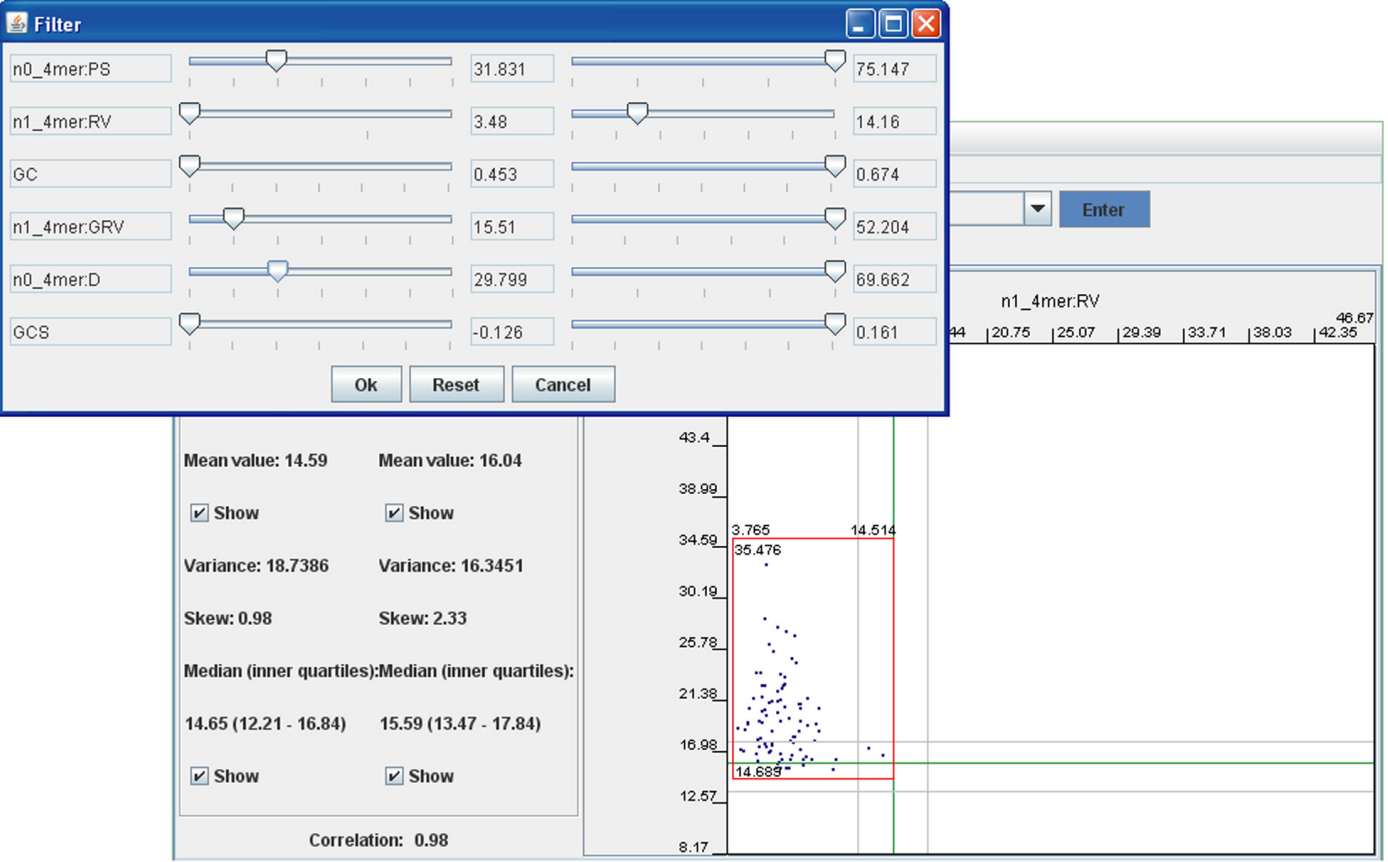
**Filtering genomic regions by multiple parameters**. Click the 'Filter' button to open a dialog as shown in the figure. Setting up border values of multiple OU statistical parameters allows more precise localisation of regions of interest.

The approach to identify inserts of foreign genomic elements by OU statistical parameters has been described recently [21]. While several algorithms allow identification of horizontally transferred genomic islands [8-13], the multiple oligomer parameters used in the SWGB even allows tentative attribution of genomic fragments (and, given the right scale, genes or gene clusters) to different functional classes using only a FASTA sequence as input. However, the emphasis of the SWGB is not primarily its annotation capability, but its ability to display the natural internal variability of genome sequences. We use *Pseudomonas putida *KT2440, a known mosaic genome with 105 genomic islands above 4000 bp in length [26] as an example. Many of these features can be visualized at a glance using the SWGB without any in depth analysis (see Fig. 2). On the 'Diagram' view the parameters n1_4mer:RV, n1_4mer:GRV and n0_4mer:D were selected for the X, Y and Z axes, respectively, as we showed previously (see Fig. 3). Plotting local relative oligomer variance (RV) against global relative variance (GRV) basically shows the effect of normalization by global mononucleotide content. The core genome is then represented on the dot plot as the positive linear correlation line where RV ≈ GRV (Fig. 3). In other words, these fragments exhibit such compositional closeness to the core genome that normalizing by local mononucleotide content does not have a different effect compared to normalizing by global content. These genomic fragments also exhibit a low distance from the genomic average; and are therefore coloured blue. Scattered dots lying peripheral to the expected strong linear correlation do not belong to the core genome and also have a higher distance from the genomic average and are hence coloured green. Using the filter settings recommended in Fig. 5, twenty one fragments were found to be genomic islands (note that while border values of OU parameters are not the same for different genomes, the grading notches of the sliders represent relative values that allows identification of homologous regions in many different genomes). For a number of reasons, many more islands were found in a similar analysis by Weinel *et al*. [26]. Firstly, the sliding window size of 8 kbp means many of the 4 kbp features from their analysis were not identified automatically. Furthermore, they were looking for all compositionally atypical regions, whereas here we restrict ourselves to horizontally transferred regions.

A known 40 kbp bacteriophage insertion [2586000–2626000] is, surprisingly, not among the genomic fragments selected in the SWGB using this filter. Although the prophage is still perceptible on the 'Gene Map' view (see a figure in the supplementary help web-pages), the OU parameters of the region do not differ markedly enough from the core sequence to be isolated automatically as a horizontally transferred region.

As the SWGB uses parameters that are based on comparison of local fragments to the global genomic average, strains with abundant insertions of homogenous DNA can confound this form of analysis. One example is the *Methanosarcina acetivorans *C2A genome which is composed of an estimated 25% of putatively horizontally acquired DNA, one of the highest amounts discovered to date [11]. As a result of these insertions, the genomic signature has been strongly influenced, resulting in a large amount of scatter and a poorly defined core genome on the plots. On the other hand, this type of analysis allows estimation of genome stability in a simple, multi parameter view (see the *Vibrio cholerae *N16961-O1-eltor example in the online help files). To conclude, filtering provides a convenient way to automatically isolate divergent genomic regions of interest. However, some regions may erroneously remain undetected due to possible amelioration of older inserts [27] or a higher level of noise in unstable genomes. However, many problematic genomic fragments can in some cases be easily attributed to functional gene categories using the SWGB 'Diagram' window (see Fig. 2).

Methodologies for discovering long modular genes have already been discussed in a previous publication [28]. Briefly, long genes display a particular tetranucleotide usage and can be discovered by plotting n0_4mer:D (X axis) versus n1_4mer:RV (Y axis). The positively linear correlated outlier fragments (towards the top right of the image) are often fragments of long genes with their characteristic repeats. An example using the gene encoding the 1.12 megadalton cell surface protein of *Synechococcus *sp. WH8102 [29] marked on the dot-plot is shown in Fig. 4. Ribosomal RNA operons (but not genes for ribosomal proteins) are characterized by extremely high pattern skew and a large distance from the core genome (Fig. 2). Thus, there is a tendency to find many genomic fragments containing rRNA genes coloured dark brown to red in the bottom right section of the 'Diagram' tab. The annotation for rRNA operons is not present in the database; therefore, these are seen in the 'Gene Map' tab as unannotated areas with high pattern skew (Fig. 2). Ribosomal proteins tend to be increasingly present at a slightly greater than average RV and above average D (see Fig. 2), which is in agreement with observations that highly expressed genes for ribosomal proteins have a highly specific codon usage compared to housekeeping genes of the organism [30]. The majority of genomic fragments form a cluster characterized by average and higher than average RV, stable OU patterns (low D) and low PS. These tend to be the core, or bulk genes and genomic regions with their typical tetranucleotide usage. Some other core sequence fragments spread from this area toward lower RV and less specific OU patterns (higher D and PS) – these are all characteristics of an unstable or randomly generated sequence [20]. These regions were found to be enriched with many hypothetical genes, prophages and transposons. (The data is not shown but is easily verified with any genome using the 'Get' button. Consider, for example, this area in the pseudogene rich *Mycobacterium leprae *TN or *Methanosarcina acetivorans *C2A genomes [11,31], and the relatively homogenous *Alcanivorax borkumensis *SK2 genome [32].) These regions were thus categorized as rich in 'vestigial' genes in contrast to the core genome regions rich in housekeeping genes (Fig. 4).

It must be stressed that with an average length of genes being around 1 kbp and overlapping sliding windows of 8 kbp, one cannot expect precise separation of housekeeping and vestigial genes by the method described above. However, when analyzing an unknown DNA sequence prior to annotation, it may be helpful to identify genomic regions enriched with a higher proportion of these so called housekeeping genes and other regions rich in vestigial genes. These tentative results should be verified with other complementary algorithms such as BLAST, gene finding and annotation techniques.

**Figure 6:**
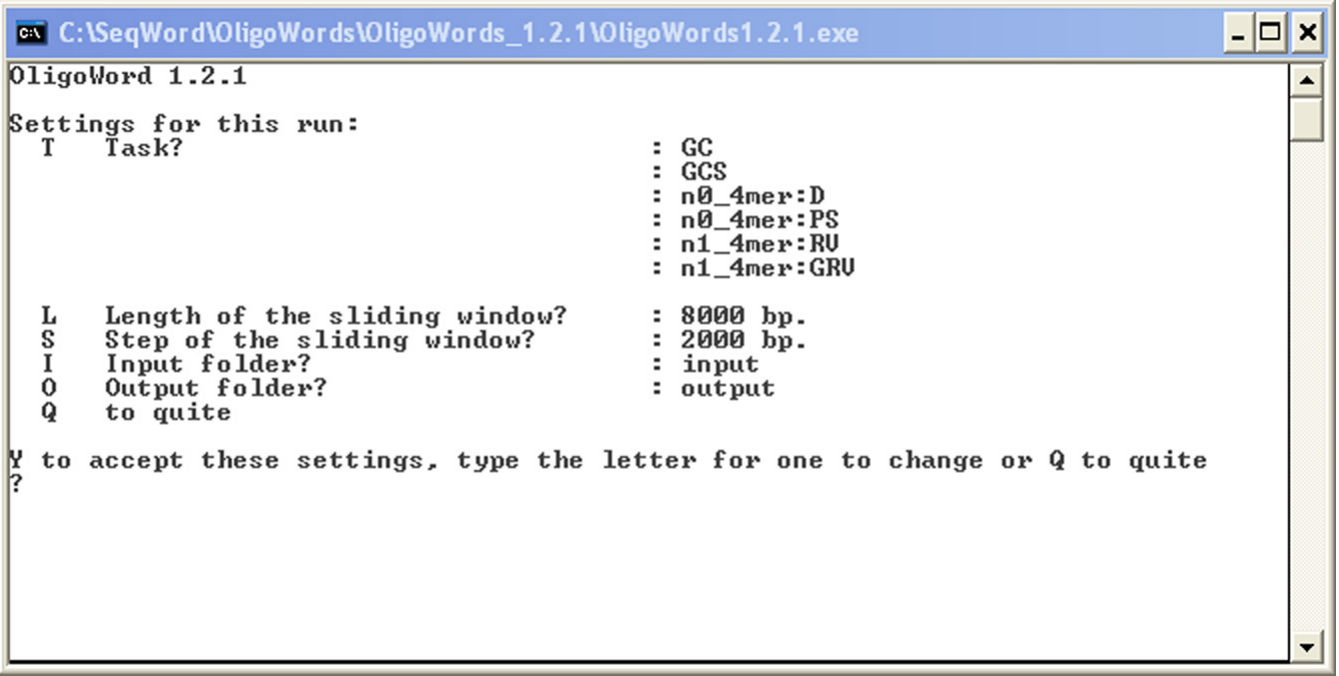
**Command-line interface of the OligoWords program**. To change the setting for the current run, type the option's letter and enter a new value as prompted. Users may change: T) the set of statistical OU parameters to be calculated for every local pattern; L) length of the sliding window; S) step of the sliding window; I) the name of the input folder that contains FASTA and/or GenBank files with source DNA sequences; and O) the name of the output folder where the result files will be stored.

The most important feature of the supplemented software available from the SWGB web-server for download is the ability to quickly and easily analyze a novel sequence on a local computer. The command-line Python program OligoWords is first used to analyse FASTA or GenBank formatted sequences. The program is available for download [33] in several packages as precompiled executable files and as Python source code. The command-line interface of the OligoWords program is shown in Fig. 6. Parameters such as oligomer length and window size can all be set depending on the sequence length and desired resolution (see Table 1 for suggestions). Since the SWGB is implemented as a Java applet, it can be run within a web browser locally. The HTML-embedded applet is available for download from the same FTP site [33] (select SeqWord_Viewer.zip). The output file from OligoWords is read into the SWGB via the 'Open' function of the 'File' menu, and the complete functionality of the online system is then available. For example, a new sequence can be analysed for ribosomal gene clusters, putative horizontally transferred elements or other regions of atypical DNA structure prior to the lengthy annotation step. A complete description of how to run the SWGB and OligoWords locally is presented in the online help files.

## Conclusion

The SWGB applies novel OU statistics to visualize and discern divergent genomic regions. It has been extensively tested in practice for large scale genome analysis [32,34], and for identification and comparison of horizontally transferred genomic islands [35]. The applet is linked to a database of pre-calculated OU patterns of bacterial genomes (1243 complete sequences, including bacterial chromosomes, plasmids and some viruses were available at the time of manuscript submission, however, new sequences are regularly being added). The SWGB allows tentative annotation of the various divergent regions and provides overviews for use in comparative genomics. Users may download the command line version of the OligoWords program to analyze their own sequences. A packaged version of the SWGB allows users to view and manipulate their OligoWords results locally using a compatible web-browser.

Although there are several readily available tools for DNA compositional analysis, genomic island identification and large scale genome analysis [36-42], the SWGB surpasses previous approaches in making use of a wider range of parameters which allow identification of divergent genomic regions and even visual tentative attribution of these DNA fragments to various categories. We have found superimposition of these parameters to be more informative than a simple GC average or a relative OU frequency deviation since they allow discrimination of divergent genomic regions (large modular genes, ribosomal RNAs, ribosomal protein clusters and the horizontally transferred genomic islands, see Fig. 2) all of which are characterized by an alternative OU composition relative to the core sequence. In addition, our approach provides some insight into the physicochemical state of the analysed DNA and the stability/state of flux of a genome as tetranucleotides exert a strong structural signal [20,21]. Consideration of flux inferred oligonucleotide usage is particularly interesting when comparing, for example, multiple replicating units of the same strain. Using the simple analysis described here, the second chromosome of *Vibrio cholerae *N16961-O1-eltor was demonstrated to be far less conserved than the first, with differences in mononucleotide content and distance from core genomic values implying a more heterogenous chromosome consistent with its role as a gene capture system [43].

Furthermore, no single oligonucleotide word size has been found to be optimal for all purposes, such as finding conserved or horizontally transferred DNA, plasmid host comparisons or testing distant homology [19]. The SWGB crucially provides the opportunity to analyse DNA sequences with various oligomer lengths and normalisation schemes. For example, genomic regions of particular interest may be multiply analysed with progressively smaller oligomer sizes to provide more detailed information on oligomer usage in individual genes.

## Availability and requirements

The SWGB applet is freely available to any researcher wishing to use it for non-commercial purposes via the Internet [22-24]. It has been tested on openSUSE 10.2, Gentoo Linux 2.6, Fedora Core 5 and Microsoft Windows XP workstations using Microsoft Internet Explorer 6.0, Maxthon 1.5.9, Mozilla Firefox 2.0, Mozilla SeaMonkey 1.1.1, Safari 3.0.4 for Mac, Konqueror 3.5.5 and 3.5.7, and Opera 9.10 browsers with Java 1.5. At the time of manuscript submission, a problem likely related to the local firewall was encountered with the Firefox browser on SUSE 10.2 and some other browsers (see 'Compatibility' link on the SWGB front page). The problem will be tackled in later releases of the SWGB. Feedback from users (addressed to the corresponding author) is very much appreciated.

## Abbreviations

SWGB: SeqWord Genome Browser; OU: oligonucleotide usage; D: distance between two oligonucleotide usage patterns; PS: oligonucleotide usage pattern skew; RV: relative variance of the oligonucleotide usage; GRV: globally normalized relative variance of the oligonucleotide usage; GC: guanine + cytosine content; GCS: guanine versus cytosine skew in DNA strands.

## Authors' contributions

HG participated in development of the MySQL database and the SWGB web-site, Python programming, applet testing; ASR contributed by Java and PHP programming; CFD participated in development and support of the SWGB web-site, Help files and the applet testing; BT participated in development and testing of the OU statistical algorithms; ONR participated in development and testing of the OU statistical algorithms, supervision of the SeqWord project and Python programming.
